# Structured psychological nursing and postpartum psychological outcomes in primiparous women: a retrospective temporal cohort study

**DOI:** 10.3389/fpsyt.2026.1857487

**Published:** 2026-09-10

**Authors:** Qiong Gao, Li-Li Zhu, Hua Guo, Zhi-Ling Zhu, Fu-Qin Zhang, Yi-Qin Wang, Bei-Jie Hou

**Affiliations:** 1 Department Delivery Room, Xinxiang Central Hospital, The Affiliated Central Hospital of Henan Medical University, Xinxiang, Henan, China; 2 School of Nursing, Henan Medical University, Xinxiang, Henan, China; 3 Department of Medical Oncology, Xinxiang Central Hospital; Xinxiang Key Laboratory of Palliative Care Skills and Nursing Clinical Practice, Xinxiang, Henan, China; 4 Department of Preventive Health Care, Xinxiang Central Hospital, Xinxiang, Henan, China

**Keywords:** anxiety, childbirth-related psychological trauma, city birth trauma scale, depression, nursing satisfaction, postpartum mothers, psychological nursing

## Abstract

**Background:**

Childbirth-related psychological symptoms may adversely affect maternal recovery and postpartum functioning. This study aimed to examine whether a structured psychological nursing program was associated with improved psychological outcomes among primiparous women.

**Methods:**

This single-center retrospective temporal-cohort study included 323 primiparous women with singleton deliveries in 2025. Women receiving routine nursing from January to June comprised the routine nursing group (n = 158), whereas those receiving routine care plus structured psychological nursing from July to December comprised the psychological nursing group (n = 165). Outcomes were assessed at 42 days postpartum using the City Birth Trauma Scale (City BiTS), Patient Health Questionnaire-9 (PHQ-9), Generalized Anxiety Disorder-7 (GAD-7), and an institutional nursing satisfaction questionnaire. Probable childbirth-related post-traumatic stress disorder (PTSD) was determined using the complete City BiTS DSM-5 and temporal-attribution algorithm.

**Results:**

The psychological nursing group had a lower mean City BiTS score than the routine nursing group (14.1 ± 7.1 vs. 18.6 ± 8.4; mean difference, −4.50; 95% CI, −6.21 to −2.79; Hedges’ g = −0.58). Probable childbirth-related PTSD occurred in 9.1% versus 17.7% of women (risk ratio, 0.51; 95% CI, 0.28–0.92). PHQ-9 and GAD-7 scores were also lower, whereas nursing satisfaction was higher. After adjustment for delivery mode and maternal age, psychological nursing remained associated with lower odds of probable PTSD (adjusted odds ratio, 0.46; 95% CI, 0.23–0.91).

**Conclusions:**

Structured psychological nursing was associated with lower postpartum trauma, depressive, and anxiety symptom burden and greater nursing satisfaction. Prospective randomized studies are required to confirm these findings.

## 1 Introduction

Childbirth is a major physiological event that can also represent a substantial psychological stressor (1, 2). The postpartum period involves rapid biological, emotional, and social transitions that may increase vulnerability to childbirth-related post-traumatic stress symptoms, depression, and anxiety. These conditions frequently coexist and may interfere with maternal recovery, caregiving confidence, and adaptation to the maternal role (3, 4). Their development is multifactorial, with obstetric complications, emergency cesarean delivery, prolonged or difficult labor, perceived loss of control, negative birth appraisal, and insufficient informational or emotional support all recognized as relevant correlates (5–7).

Accordingly, increasing attention has been directed toward early identification and low-burden psychological support embedded within routine maternity care (8). The City Birth Trauma Scale (City BiTS), which is structured around the Diagnostic and Statistical Manual of Mental Disorders, Fifth Edition criteria, provides a childbirth-specific measure of post-traumatic stress symptoms (9). The City BiTS has demonstrated a stable factorial structure, high internal consistency, test–retest reliability, and satisfactory convergent, divergent, and predictive validity in postpartum populations, supporting its use in both clinical and research settings. At the same time, emerging evidence suggests that accessible psychosocial strategies integrated into usual care pathways may be associated with lower postpartum psychological distress (10, 11). Psychological nursing is well suited to this context because it can combine early assessment, supportive communication, childbirth-related education, cognitive guidance, relaxation training, and family involvement during routine obstetric care (12, 13). Such a structured approach may address modifiable contributors to postpartum distress before symptoms become persistent or severe. However, although recent observational studies have further characterized the prevalence and correlates of childbirth-related psychological trauma, evidence regarding the association between structured psychological nursing and postpartum psychological outcomes remains limited, particularly in real-world obstetric settings (14, 15).

Therefore, this study examined the association of structured psychological nursing integrated with routine obstetric care with childbirth-related post-traumatic stress symptoms, probable childbirth-related post-traumatic stress disorder, depressive and anxiety symptoms, and nursing satisfaction at 42 days postpartum among primiparous women.

## 2 Methods

### 2.1 Study design

This single-center retrospective temporal-cohort study included primiparous women who delivered at our institution between January and December 2025. Nursing exposure was determined by the standard clinical practice in effect during consecutive calendar periods rather than by research assignment. Women who delivered between January and June 2025 and received routine obstetric nursing care constituted the routine nursing group, whereas those who delivered between July and December 2025 and received routine care supplemented by the structured psychological nursing program constituted the psychological nursing group. Participants were not randomized, and the investigators did not assign or modify the nursing strategy for research purposes. Women were eligible if they were primiparous, had a singleton delivery during the study period, had sufficient language and cognitive capacity to complete the routine psychological assessments, and had the key demographic, obstetric, and neonatal data required for eligibility assessment. Women were excluded before the 42-day follow-up if they had a documented history of a psychiatric disorder, severe cognitive or communication impairment that precluded questionnaire completion, severe pregnancy complications or serious postpartum medical conditions that precluded standardized psychological assessment or required intensive care, a major adverse perinatal event such as stillbirth or neonatal death, or missing key clinical data required for eligibility determination. Women who otherwise met the eligibility criteria but did not complete the routine 42-day postpartum assessment were not included in the final analytic cohort. Missing or incomplete intervention-delivery records alone did not result in exclusion from the primary outcome analysis; implementation analyses were based on available contemporaneous nursing documentation. Informed consent was obtained from all subjects. The study was reviewed and approved by the ethics committee of our hospital. All methods were performed in accordance with relevant guidelines and regulations and with the ethical principles of the Declaration of Helsinki. All data were anonymized before analysis to protect participant privacy.

### 2.2 Nursing interventions

#### 2.2.1 Routine nursing care

Women in the routine nursing group received the standard obstetric nursing care used at the institution from January to June 2025. Routine care included admission education, maternal and fetal monitoring, labor and pain-management support, postoperative or postpartum observation, breastfeeding and newborn-care guidance, discharge education, and referral for additional assessment when clinically indicated. No structured multicomponent psychological nursing protocol was routinely implemented during this period.

#### 2.2.2 Structured psychological nursing program

From July to December 2025, women received routine obstetric nursing care together with a structured psychological nursing program implemented under a standardized departmental protocol (version 1.0). The same protocol was used throughout this period without substantive changes to its content, eligibility criteria, or delivery schedule. It represented a standardized institutional clinical pathway rather than a separately published manualized psychotherapy program. The program included predefined core components and individually tailored components. Core components comprised psychological assessment within 2 h of admission, childbirth-related psychoeducation before active labor or cesarean delivery, emotional validation and supportive communication, guided relaxation, family-support guidance when an appropriate caregiver was available, intrapartum emotional support for women who entered labor, and psychological review at 24–72 h postpartum. Emotional support was intended to include at least two documented contacts. The planned minimum exposure was three contacts, with a target cumulative duration of at least 60 min. Tailored components were provided according to documented clinical needs and included cognitive intervention for maladaptive beliefs, catastrophic thinking, or marked fear of childbirth; brief mindfulness-based exercises for elevated anxiety or distress; additional psychological support for persistent or worsening symptoms before discharge or during routine postpartum follow-up; and specialist psychological or psychiatric referral for severe symptoms, marked functional impairment, self-harm concerns, or other clinically significant concerns. When additional support or referral was provided at the 42-day follow-up visit, the City BiTS, PHQ-9, GAD-7, and nursing satisfaction assessments were completed beforehand. Accordingly, not all women received every tailored component. The program was delivered by 12 registered obstetric nurses, eight of whom had at least 5 years of obstetric nursing experience. Before implementation, all nurses completed an 8-h theoretical workshop, a 2-h simulated practice session, and a standardized competency assessment. Intervention components, contact frequency, documented duration, nurse assignment, and referral were recorded in routine clinical and nursing records. Because the study was retrospective, intervention implementation and fidelity were evaluated using contemporaneous nursing checklists, progress notes, contact logs, referral records, and routine quality-control documentation rather than prospective research monitoring. Routine quality control included independent review of at least 20% of intervention records. Component completion and documented intervention duration were also summarized by nurse to assess between-nurse variability. Missing implementation records were reported and were not imputed.

### 2.3 Data sources and retrospective data collection

Demographic, obstetric, neonatal, nursing, and follow-up data were retrospectively extracted from the electronic medical record, obstetric delivery records, operative reports, nursing progress notes, standardized intervention checklists, nursing quality-control records, and postpartum follow-up records.

The extracted baseline variables included maternal age, gestational age at delivery, educational level, pre-delivery body mass index, gestational diabetes mellitus, hypertensive disorders of pregnancy, other pregnancy-related complications, delivery mode, entry into labor, labor duration, labor analgesia, perineal laceration or episiotomy, postpartum hemorrhage, neonatal birth weight, 1-min Apgar score, and neonatal intensive care unit admission.

Delivery mode was classified as vaginal, elective cesarean, or emergency cesarean delivery based on contemporaneous obstetric, operative, and scheduling records. Elective cesarean delivery was defined as a procedure documented as planned, whereas emergency cesarean delivery was defined as unplanned or performed on an emergency basis. Classification followed the urgency recorded at the time of care rather than the documented obstetric indication alone. Women who entered labor or developed an intrapartum condition before a previously planned procedure retained the urgency classification recorded in the operative record. Cesarean indications and intrapartum conditions, including fetal distress, labor arrest, and failed induction, were extracted separately. Labor duration and analgesia were evaluated only among women who entered labor, and perineal laceration or episiotomy only among women who delivered vaginally.

For women in the psychological nursing group, implementation data included the intervention components for which the woman was eligible, actual component completion, dates and numbers of contacts, documented contact duration, cumulative intervention duration, the nurse delivering the intervention, completion of all applicable core components, additional psychological support, and psychological or psychiatric referral.

### 2.4 Postpartum follow-up and assessor masking

Psychological outcomes were assessed at the routine 42-day postpartum follow-up. Assessments were conducted by four designated follow-up nurses who had not participated in delivery of the psychological nursing program. These assessors were instructed not to review intervention records before questionnaire administration, and intervention records were not available to them during the assessment. However, because group membership was determined by calendar period, complete masking to the nursing strategy could not be guaranteed, and masking integrity was not formally evaluated. The City Birth Trauma Scale, Patient Health Questionnaire-9, Generalized Anxiety Disorder-7 scale, and nursing satisfaction questionnaire were completed before any additional counseling or specialist referral provided during the same follow-up visit.

### 2.5 Outcome measures

#### 2.5.1 Primary outcomes

The primary outcomes were the City Birth Trauma Scale (City BiTS) total symptom score and the presence of probable childbirth-related post-traumatic stress disorder at 42 days postpartum. The City BiTS is a 29-item self-report instrument structured according to the Diagnostic and Statistical Manual of Mental Disorders, Fifth Edition framework for PTSD following childbirth. The total symptom score was calculated by summing items Q3–Q22, with a possible range of 0–60; higher scores indicated greater symptom severity (16).

Probable PTSD was determined using the complete DSM-5 symptom algorithm rather than a single total-score cutoff. Criterion A was considered fulfilled when Q1 or Q2 was answered “yes.” Re-experiencing criterion B required at least one item among Q3–Q7 scored ≥1; avoidance criterion C required at least one item among Q8–Q9 scored ≥1; negative cognitions and mood criterion D required at least two items among Q10–Q16 scored ≥1; and hyperarousal criterion E required at least two items among Q17–Q22 scored ≥1. The duration criterion was fulfilled when Q26 was scored ≥1, and the distress or impairment criterion was fulfilled when Q27 or Q28 was scored ≥1. Participants with Q29 scored ≥1 were not classified as probable PTSD because symptoms could be attributable to medication, alcohol, drugs, or physical illness. Items Q23–Q24 were used to record the dissociative subtype but were not required for the core classification.

Q25 was used exclusively to establish the temporal relationship between symptom onset and the index childbirth and was not treated as an additional DSM-5 symptom criterion. Probable childbirth-related PTSD was assigned only when the core DSM-5 algorithm was fulfilled and Q25 did not indicate that the symptoms predated the index childbirth. Cases with symptoms predating childbirth or with missing or uncertain onset information were not assigned childbirth-related attribution in the primary analysis. Complete responses to all items required for the DSM-5 and temporal-attribution algorithms were required for classification.

The City BiTS total symptom score and the algorithm-based binary classification were analyzed as separate outcomes. Because the binary outcome was derived from a self-report instrument rather than a clinician-administered diagnostic interview, it was termed probable childbirth-related PTSD and was not considered a confirmed clinical diagnosis.

#### 2.5.2 Secondary psychological outcomes

Depressive symptoms were assessed using the 9-item Patient Health Questionnaire (PHQ-9) (17), and anxiety symptoms were assessed using the 7-item Generalized Anxiety Disorder scale (GAD-7) (18). Higher scores indicated more severe symptoms. PHQ-9 and GAD-7 scores were analyzed as continuous variables. A PHQ-9 score ≥10 was used to define a positive depressive-symptom screen, and a GAD-7 score ≥10 was used to define a positive anxiety-symptom screen. These thresholds represented screening classifications and were not interpreted as clinician-confirmed psychiatric diagnoses.

#### 2.5.3 Nursing satisfaction

Nursing satisfaction was assessed using the Institutional Nursing Satisfaction Questionnaire, a 20-item instrument developed and routinely used by the hospital nursing quality-management department. The 2024 obstetric nursing version covers communication and respect, technical nursing care, emotional support, health education, and continuity and responsiveness. Each item is scored on a five-point Likert scale from 1 (very dissatisfied) to 5 (very satisfied), producing a total score from 20 to 100; higher scores indicate greater satisfaction.

The questionnaire was administered at the 42-day follow-up after completion of the City BiTS, PHQ-9, and GAD-7. Internal consistency was evaluated using Cronbach’s α in the overall cohort and separately by nursing group. Floor and ceiling effects were also examined. Because the instrument had not undergone published external validation and no minimal clinically important difference had been established, nursing satisfaction was treated as an exploratory patient-reported care-experience outcome rather than a validated clinical endpoint.

### 2.6 Statistical analysis

All statistical analyses were performed using IBM SPSS Statistics, version 30.0 (IBM Corp., Armonk, NY, USA). Continuous variables were summarized as mean ± standard deviation or median with interquartile range, as appropriate. Distributional assumptions were evaluated using histograms and Q–Q plots, and homogeneity of variance was assessed using Levene’s test. Between-group comparisons were performed using Student’s t test, Welch’s t test when variances were unequal, or the Mann–Whitney U test for markedly skewed data. Categorical variables were compared using Pearson’s chi-square test without continuity correction or Fisher’s exact test when expected cell counts were small. Baseline balance was additionally evaluated using absolute standardized mean differences. For continuous outcomes, mean differences and Hedges’ g values were reported with 95% confidence intervals. Binary outcomes were summarized using relative risks, absolute risk differences, and 95% confidence intervals; Cohen’s h was additionally calculated for probable childbirth-related PTSD. Internal consistency of the City BiTS total symptom scale and its birth-related and general symptom subscales was evaluated using Cronbach’s α. Missingness was assessed separately for baseline characteristics, obstetric variables, item-level psychological scale data, nursing satisfaction data, delivery-mode classification, and intervention-delivery records. Reasons for missingness and evaluable sample sizes were summarized by nursing group. No statistical imputation was performed. Primary and secondary outcome analyses were based on complete cases, whereas intervention implementation and fidelity summaries used available contemporaneous documentation. Available baseline characteristics were compared between included and excluded women to explore potential selection bias. Probable childbirth-related PTSD was examined using multivariable logistic regression including nursing strategy, elective and emergency cesarean delivery, and maternal age. Adjusted odds ratios with 95% confidence intervals were reported. The model included 43 events and four predictor parameters. Predictor correlations, tolerance, and variance inflation factors were used to assess multicollinearity, and Firth penalized logistic regression was performed as a sensitivity analysis. Spearman correlation was used to examine relationships among City BiTS, PHQ-9, and GAD-7 scores. Subgroup analyses, interaction tests, and elective-versus-emergency cesarean comparisons were considered exploratory. All tests were two-sided, with P < 0.05 considered statistically significant.

## 3 Results

### 3.1 Participant selection and follow-up completion

**Figure 1:**
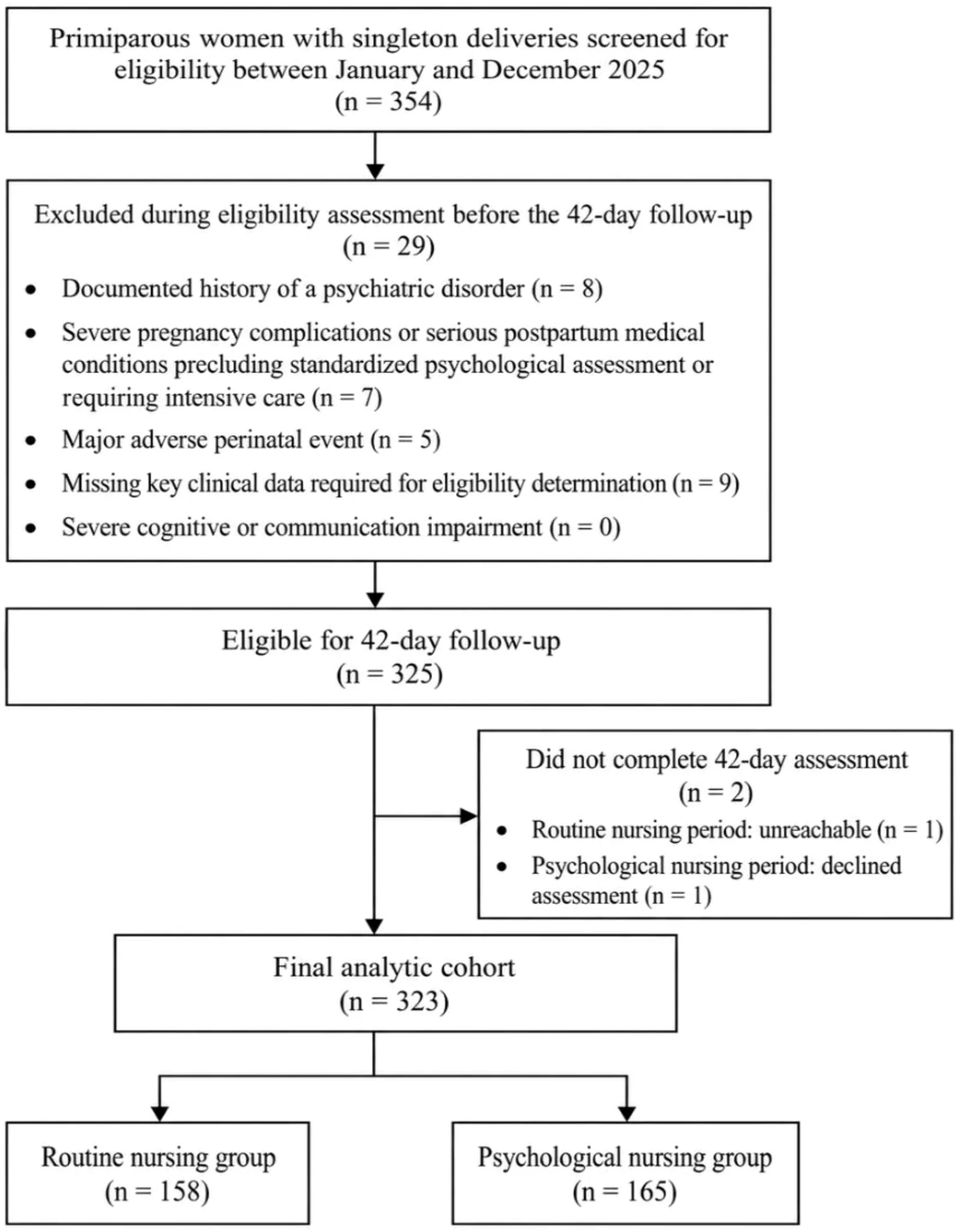
Participant selection and study flow.

Participant screening, exclusions, follow-up completion, and final group allocation are summarized in **Figure 1**. All included women had complete City BiTS, PHQ-9, GAD-7, and nursing satisfaction data at 42 days postpartum (**Supplementary Table 1**). Available baseline data for the 31 women not included in the final analytic cohort were compared with those of the included participants. No substantial differences were observed for most available characteristics, although the estimates were imprecise because of incomplete data. The largest absolute standardized mean difference was 0.221 for gestational age at delivery (**Supplementary Table 2**).

### 3.2 Baseline characteristics

The demographic, obstetric, and neonatal characteristics of the two study groups are presented in **Table 1**. The groups were similar with respect to maternal age, gestational age, educational level, pre-delivery body mass index, pregnancy-related complications, entry into labor, labor analgesia, perineal laceration or episiotomy, neonatal birth weight, 1-min Apgar score, postpartum hemorrhage, and neonatal intensive care unit admission. Delivery mode was also similarly distributed between the groups: vaginal delivery occurred in 70.3% of women in the routine nursing group and 72.1% in the psychological nursing group, elective cesarean delivery in 11.4% and 12.1%, and emergency cesarean delivery in 18.4% and 15.8%, respectively. All absolute standardized mean differences were below 0.20.

**Table 1:** Baseline demographic, obstetric, and delivery characteristics.

Variable	Routine nursing group (n = 158)	Psychological nursing group (n = 165)	Absolute SMD	P value
Age, years	28.7 ± 4.1	28.9 ± 4.3	0.048	0.669
Gestational age at delivery, weeks	39.1 ± 1.2	39.2 ± 1.1	0.087	0.436
Educational level, n (%)				0.844
High school or below	48 (30.4)	46 (27.9)	0.055	
Junior college	86 (54.4)	91 (55.2)	0.014	
Bachelor’s degree or above	24 (15.2)	28 (17.0)	0.048	
Pre-delivery BMI, kg/m²	26.4 ± 3.5	26.1 ± 3.3	0.088	0.429
Gestational diabetes mellitus, n (%)	18 (11.4)	16 (9.7)	0.055	0.620
Hypertensive disorders of pregnancy, n (%)	14 (8.9)	13 (7.9)	0.035	0.750
Other pregnancy-related complications, n (%)	12 (7.6)	11 (6.7)	0.036	0.746
Mode of delivery, n (%)				0.821
Vaginal delivery	111 (70.3)	119 (72.1)	0.041	
Elective cesarean delivery	18 (11.4)	20 (12.1)	0.023	
Emergency cesarean delivery	29 (18.4)	26 (15.8)	0.069	
Entered labor before delivery, n (%)	143 (90.5)	148 (89.7)	0.027	0.808
Duration of labor among women entering labor, h	9.8 ± 3.1 (n = 143)	9.4 ± 3.0 (n = 148)	0.131	0.264
Labor analgesia among women entering labor, n/N (%)	72/143 (50.3)	81/148 (54.7)	0.088	0.454
Perineal laceration or episiotomy among vaginal deliveries, n/N (%)	94/111 (84.7)	103/119 (86.6)	0.053	0.686
Neonatal birth weight, g	3325 ± 418	3362 ± 405	0.090	0.420
1-min Apgar score	9.2 ± 0.7	9.3 ± 0.6	0.154	0.170
Postpartum hemorrhage, n (%)	10 (6.3)	8 (4.8)	0.064	0.562
NICU admission, n (%)	7 (4.4)	6 (3.6)	0.040	0.717

BMI, body mass index; NICU, neonatal intensive care unit; SMD, standardized mean difference.

### 3.3 Implementation of the psychological nursing program

The psychological nursing program was delivered according to a written departmental protocol by 12 registered obstetric nurses who had completed standardized theoretical training, simulated practice, and competency assessment. Initial psychological assessment and childbirth-related psychoeducation were documented for all 165 women in the psychological nursing group. At least two emotional-support contacts were documented for 163 women (98.8%). Guided relaxation training was completed by 159 of 162 women with evaluable documentation (98.1%), family-support intervention by 151 of 160 eligible women (94.4%), and intrapartum emotional support by 143 of 148 women who entered labor (96.6%). A postpartum psychological review at 24–72 h was completed by 160 women.

Among the 157 women with complete component-level records, 148 (94.3%) completed all applicable core intervention components. Among the 158 women with complete contact logs, the median number of intervention contacts was 4 (IQR, 3–5), and the median cumulative documented intervention duration was 95 min (IQR, 80–120 min). Six women met the prespecified criteria for specialist referral, and all six were referred for psychological or psychiatric assessment. An independent audit of 33 intervention records found that 30 (90.9%) met all assessed protocol criteria, while three contained minor deviations. The 42-day questionnaires were administered before any additional counseling or referral at the same visit. Follow-up assessors had not participated in intervention delivery, although complete masking to the calendar-based group allocation could not be guaranteed (**Table 2**; **Supplementary Table 3**).

**Table 2:** Delivery and implementation of the psychological nursing intervention.

Implementation indicator	Prespecified requirement	Actual delivery	Documentation source	Missing documentation
Written intervention protocol	One standardized departmental protocol applicable throughout the intervention period	One written protocol, version 1.0, was used throughout the intervention period from July to December 2025	Departmental standard operating procedure and protocol archive	0
Protocol modification during implementation	Any modification to the intervention protocol was required to be documented and approved	No substantive modification to the intervention content, eligibility criteria, or delivery schedule was documented during the study period	Protocol amendment log and nursing quality-control records	0
Core and tailored intervention components	Initial assessment, psychoeducation, emotional support, relaxation training, eligible family support, and postpartum review were designated as core components; cognitive intervention, mindfulness exercises, additional support, and specialist referral were delivered according to prespecified clinical criteria	Core components were offered to all eligible mothers. Tailored components were delivered only when the corresponding eligibility criteria were documented	Intervention protocol, eligibility checklist, and nursing progress records	0
Number and qualifications of intervention nurses	Registered obstetric nurses who had completed standardized intervention training	The intervention was delivered by 12 registered obstetric nurses; 8/12 had at least 5 years of obstetric nursing experience	Staffing roster and personnel files	0
Training duration	Completion of standardized theoretical and practical training before intervention delivery	All 12 nurses completed an 8-h theoretical workshop and 2-h simulated practice session	Training attendance records	0
Competency assessment	Nurses were required to pass a standardized competency assessment before delivering the intervention	All 12 nurses passed the competency assessment before participating in intervention delivery	Competency assessment forms	0
Intervention caseload per nurse	The intervention workload was distributed across the 12 trained nurses	The median caseload was 14 mothers per nurse, with a range of 12–15	Nurse assignment log	0
Initial psychological assessment	Assessment within 2 h after admission	Completed for 165/165 mothers (100.0%)	Admission psychological assessment form	0
Childbirth-related psychoeducation	At least one structured session before delivery	Completed for 165/165 mothers (100.0%)	Nursing intervention checklist	0
Emotional validation and supportive communication	At least two documented supportive contacts	At least two contacts were documented for 163/165 mothers (98.8%); the remaining 2 mothers had one documented contact	Nursing progress notes	0
Guided relaxation training	At least one guided breathing-regulation or progressive muscle-relaxation session	Among 162 mothers with evaluable documentation, 159 completed the component (98.1%) and 3 had documented noncompletion; records were unavailable for another 3 mothers	Relaxation-training checklist	3
Family-support intervention	One structured contact with a spouse or primary caregiver when an appropriate support person was available	Of 160 eligible mothers, 151 completed the component (94.4%), 4 had documented noncompletion, and 5 had incomplete records; 5 mothers had no available spouse or primary caregiver and were therefore not eligible	Family-support record	5
Intrapartum emotional support	Emotional support during labor for mothers who entered labor	Of 148 eligible mothers who entered labor, 143 completed the component (96.6%), 3 had documented noncompletion, and 2 had incomplete records; 17 mothers did not enter labor and were therefore not eligible	Labor nursing records	2
Postpartum psychological review at 24–72 h	One structured psychological review before discharge	The review was completed for 160 mothers, 3 had documented noncompletion, and 2 had incomplete records	Postpartum psychological review form	2
Completion of all core intervention components	Completion of the initial assessment, psychoeducation, emotional support, relaxation training, eligible family support, and postpartum psychological review	Among 157 mothers with complete component-level documentation, 148 completed all applicable core components (94.3%) and 9 did not; component-level documentation was incomplete for 8 mothers	Composite intervention-fidelity checklist	8
Number of intervention contacts	At least three intervention contacts per mother	Among 158 mothers with complete contact logs, the median number of contacts was 4 (IQR, 3–5)	Intervention contact log	7
Cumulative intervention duration	Target cumulative duration of at least 60 min per mother	Among 158 mothers with complete contact logs, the median cumulative duration was 95 min (IQR, 80–120 min)	Intervention contact log	7
Cognitive intervention	Delivered to mothers with documented maladaptive beliefs, catastrophic thinking, or marked fear of childbirth	Of 67 eligible mothers, 61 completed the component (91.0%), 2 had documented noncompletion, and 4 had incomplete records; 98 mothers did not meet the eligibility criteria	Risk assessment and cognitive-intervention form	4
Brief mindfulness exercise	Delivered to mothers with elevated anxiety or distress or according to participant preference	Of 104 eligible mothers, 89 completed the component (85.6%), 9 had documented noncompletion, and 6 had incomplete records; 61 mothers did not meet the eligibility criteria	Mindfulness exercise record	6
Additional psychological support	Delivered to mothers with persistent or worsening psychological distress	Of 31 eligible mothers, 29 received additional psychological support (93.5%) and 2 had incomplete records; 134 mothers did not meet the eligibility criteria	Postpartum follow-up record	2
Psychological or psychiatric referral	Referral when severe psychological symptoms, marked functional impairment, or safety concerns were identified	All 6 mothers meeting the prespecified referral criteria were referred for specialist psychological or psychiatric assessment (100.0%)	Specialist referral record	0
Independent fidelity audit	Independent review of at least 20% of intervention records	A total of 33/165 records (20.0%) were independently audited; 30/33 audited records (90.9%) met all assessed protocol criteria, whereas 3 contained minor protocol deviations	Nursing quality-control audit	0
Nurse-level core-component completion	No substantial variation in core-component completion between intervention nurses	The median nurse-level core-component completion rate was 92.3%, with a range of 90.9%–100.0% among participants with complete fidelity documentation	Nurse-specific fidelity summary	8 participant records
Nurse-level cumulative intervention duration	Comparable intervention intensity across nurses	Nurse-specific median cumulative intervention duration ranged from 88 to 103 min	Nurse-specific intervention contact logs	7 participant records
Independence of 42-day outcome assessors	Follow-up assessors should not have participated in delivery of the psychological nursing intervention	All 323 follow-up assessments were completed by 4 designated follow-up nurses who were not involved in intervention delivery	Follow-up staffing log	0
Masking of 42-day outcome assessors	Assessors were instructed not to review intervention records before questionnaire administration	Intervention records were not available to assessors during questionnaire administration; however, complete masking to the calendar-based group allocation could not be guaranteed	Follow-up procedure and electronic access records	Masking integrity was not formally evaluated
Sequence of 42-day outcome assessment and subsequent support	City BiTS, PHQ-9, GAD-7, and nursing satisfaction assessments were required to be completed before any additional counseling or referral at the same visit	The required sequence was documented for 323/323 participants (100.0%)	Questionnaire timestamps and postpartum follow-up notes	0

City BiTS, City Birth Trauma Scale; GAD-7, 7-item Generalized Anxiety Disorder scale; IQR, interquartile range; PHQ-9, 9-item Patient Health Questionnaire.

### 3.4 Primary outcomes

At 42 days postpartum, the mean City BiTS total symptom score was 18.6 ± 8.4 in the routine nursing group and 14.1 ± 7.1 in the psychological nursing group. The mean between-group difference was −4.50 points (95% CI, −6.21 to −2.79), corresponding to a Hedges’ g of −0.58 (95% CI, −0.80 to −0.36). In the current cohort, Cronbach’s α was 0.92 for the City BiTS total symptom scale, 0.90 for the birth-related symptom subscale, and 0.88 for the general symptom subscale, indicating satisfactory internal consistency.

The complete City BiTS DSM-5 algorithm identified core probable PTSD in 28 of 158 women (17.7%) in the routine nursing group and 15 of 165 women (9.1%) in the psychological nursing group. Among all 43 women who fulfilled the core algorithm, Q25 indicated that the reported symptoms began after the index childbirth; none reported symptoms predating childbirth, and none had missing or uncertain onset information. Consequently, all 43 core cases also met the study definition of probable childbirth-related PTSD.

Probable childbirth-related PTSD was therefore identified in 28 women (17.7%) in the routine nursing group and 15 women (9.1%) in the psychological nursing group. The relative risk was 0.51 (95% CI, 0.28–0.92), and the absolute risk difference was −8.63 percentage points (95% CI, −16.03 to −1.24). The City BiTS total symptom score and the algorithm-based classification were analyzed as separate outcomes; the binary classification was not derived from a single total-score cutoff. Because this classification was based on a self-report screening instrument rather than a clinician-administered diagnostic interview, it represented probable childbirth-related PTSD rather than a confirmed clinical diagnosis (**Table 3**; **Supplementary Table 4**).

**Table 3:** Primary outcomes at 42 days postpartum.

Outcome	Routine nursing group (n = 158)	Psychological nursing group (n = 165)	Effect estimate	95% CI	Standardized effect size	P value
City BiTS total symptom score	18.6 ± 8.4	14.1 ± 7.1	Mean difference = −4.50	−6.21 to −2.79	Hedges’ g = −0.58 (−0.80 to −0.36)	<0.001
Probable childbirth-related PTSD according to the complete City BiTS DSM-5 and temporal-attribution algorithm, n (%)	28 (17.7)	15 (9.1)	RR = 0.51	0.28 to 0.92	Cohen’s h = −0.26	0.022
Absolute risk difference for probable PTSD	17.70%	9.10%	−8.63 percentage points	−16.03 to −1.24	—	0.022

CI, confidence interval; City BiTS, City Birth Trauma Scale; DSM-5, Diagnostic and Statistical Manual of Mental Disorders, Fifth Edition; PTSD, post-traumatic stress disorder; RR, risk ratio.

### 3.5 Secondary psychological and nursing outcomes

The mean PHQ-9 score was 6.3 ± 3.8 in the routine nursing group and 4.8 ± 3.2 in the psychological nursing group, with a mean difference of −1.50 points (95% CI, −2.27 to −0.73; Hedges’ g, −0.43, 95% CI, −0.65 to −0.21). A positive depressive-symptom screen, defined as a PHQ-9 score ≥10, was observed in 34 women (21.5%) and 20 women (12.1%), respectively (RR, 0.56; 95% CI, 0.34–0.94; absolute risk difference, −9.40 percentage points; 95% CI, −17.51 to −1.28).

The mean GAD-7 score was 5.6 ± 3.4 in the routine nursing group and 4.2 ± 2.9 in the psychological nursing group. The mean difference was −1.40 points (95% CI, −2.09 to −0.71), with a Hedges’ g of −0.44 (95% CI, −0.66 to −0.22). A positive anxiety-symptom screen, defined as a GAD-7 score ≥10, occurred in 32 women (20.3%) and 18 women (10.9%), respectively (RR, 0.54; 95% CI, 0.32–0.92; absolute risk difference, −9.34 percentage points; 95% CI, −17.21 to −1.48).

The mean nursing satisfaction score was 86.9 ± 7.5 in the routine nursing group and 91.6 ± 6.8 in the psychological nursing group, corresponding to a mean difference of 4.70 points (95% CI, 3.13–6.27; Hedges’ g, 0.66, 95% CI, 0.43–0.88). Questionnaire completion was 100% in both groups. The institutional satisfaction questionnaire showed an overall Cronbach’s α of 0.91, with group-specific values of 0.89 and 0.92. Because the instrument had not undergone external validation and no minimum clinically important difference had been established, the satisfaction findings were considered exploratory (**Table 4**; **Supplementary Table 5**).

**Table 4:** Secondary psychological and nursing outcomes at 42 days postpartum.

Outcome	Routine nursing group (n = 158)	Psychological nursing group (n = 165)	Effect estimate	95% CI	Standardized effect size or risk difference	P value
PHQ-9 total score	6.3 ± 3.8	4.8 ± 3.2	Mean difference = −1.50	−2.27 to −0.73	Hedges’ g = −0.43 (−0.65 to −0.21)	<0.001
Positive depressive-symptom screen, PHQ-9 ≥10, n (%)	34 (21.5)	20 (12.1)	RR = 0.56	0.34 to 0.94	Risk difference = −9.40 percentage points (−17.51 to −1.28)	0.024
GAD-7 total score	5.6 ± 3.4	4.2 ± 2.9	Mean difference = −1.40	−2.09 to −0.71	Hedges’ g = −0.44 (−0.66 to −0.22)	<0.001
Positive anxiety-symptom screen, GAD-7 ≥10, n (%)	32 (20.3)	18 (10.9)	RR = 0.54	0.32 to 0.92	Risk difference = −9.34 percentage points (−17.21 to −1.48)	0.020
Nursing satisfaction score	86.9 ± 7.5	91.6 ± 6.8	Mean difference = 4.70	3.13 to 6.27	Hedges’ g = 0.66 (0.43 to 0.88)	<0.001
Satisfaction questionnaire completion, n/N (%)	158/158 (100.0)	165/165 (100.0)	Risk difference = 0.00 percentage points	−2.28 to 2.37	—	Not tested
Internal consistency of the satisfaction questionnaire	Cronbach’s α = 0.89	Cronbach’s α = 0.92	Overall Cronbach’s α = 0.91	—	—	Not applicable

CI, confidence interval; GAD-7, 7-item Generalized Anxiety Disorder scale; PHQ-9, 9-item Patient Health Questionnaire; RR, risk ratio.

### 3.6 Multivariable analysis

Pairwise correlations among the predictors included in the parsimonious model were weak, with absolute correlation coefficients ranging from 0.011 to 0.165. The tolerance values ranged from 0.969 to 0.998, and the variance inflation factors ranged from 1.00 to 1.03, indicating no evidence of important multicollinearity among nursing strategy, elective cesarean delivery, emergency cesarean delivery, and maternal age (**Supplementary Table 6**).

A parsimonious multivariable logistic regression model was fitted with probable childbirth-related PTSD as the dependent variable. The model included nursing strategy, delivery mode categorized as vaginal, elective cesarean, or emergency cesarean delivery, and maternal age. After adjustment for delivery mode and maternal age, membership in the psychological nursing group was associated with lower odds of probable childbirth-related PTSD compared with membership in the routine nursing group (adjusted OR, 0.46; 95% CI, 0.23–0.91; P = 0.025).

Emergency cesarean delivery was associated with higher odds of probable childbirth-related PTSD compared with vaginal delivery (adjusted OR, 2.97; 95% CI, 1.40–6.32; P = 0.005). The estimate for elective cesarean delivery was less precise and did not provide clear evidence of an association with the outcome (adjusted OR, 1.81; 95% CI, 0.67–4.86; P = 0.241). Maternal age was also not clearly associated with probable childbirth-related PTSD in the adjusted model (adjusted OR per 1-year increase, 1.05; 95% CI, 0.97–1.13; P = 0.275) (**Table 5**).

**Table 5:** Parsimonious multivariable logistic regression for probable childbirth-related PTSD.

Predictor	Comparison/reference	Adjusted OR	95% CI	P value
Nursing strategy	Psychological nursing vs. routine nursing	0.46	0.23–0.91	0.025
Mode of delivery	Elective cesarean delivery vs. vaginal delivery	1.81	0.67–4.86	0.241
Mode of delivery	Emergency cesarean delivery vs. vaginal delivery	2.97	1.40–6.32	0.005
Maternal age	Per 1-year increase	1.05	0.97–1.13	0.275

CI, confidence interval; OR, odds ratio; PTSD, post-traumatic stress disorder.

The model included 323 women and 43 probable childbirth-related PTSD events. Four predictor parameters were estimated, corresponding to approximately 10.8 events per parameter. Vaginal delivery was the reference category. The observed numbers of events were 23/230 among vaginal deliveries, 6/38 among elective cesarean deliveries, and 14/55 among emergency cesarean deliveries.

In the Firth penalized logistic regression sensitivity analysis, the association estimates were similar to those obtained from the conventional logistic regression model. Psychological nursing remained associated with lower odds of probable childbirth-related PTSD (penalized adjusted OR, 0.47; 95% profile-likelihood CI, 0.24–0.91; P = 0.025), whereas emergency cesarean delivery remained associated with higher odds of the outcome (penalized adjusted OR, 2.77; 95% profile-likelihood CI, 1.30–5.93; P = 0.008). The estimates for elective cesarean delivery and maternal age remained imprecise and were not statistically significant (**Supplementary Table 7**).

### 3.7 Correlations among psychological symptom measures

City BiTS, PHQ-9, and GAD-7 scores were positively correlated at 42 days postpartum. The Spearman correlation coefficient was 0.58 (95% CI, 0.50–0.65) between City BiTS and PHQ-9 scores, 0.61 (95% CI, 0.53–0.68) between City BiTS and GAD-7 scores, and 0.72 (95% CI, 0.66–0.77) between PHQ-9 and GAD-7 scores. These findings showed moderate-to-strong positive correlations among the three related but distinct symptom domains and did not establish temporal or causal relationships among them (**Supplementary Table 8**).

### 3.8 Exploratory subgroup analyses

Exploratory subgroup analyses were conducted according to labor analgesia, the presence of gestational diabetes mellitus and/or hypertensive disorders of pregnancy, and educational level. The subgroup-specific odds ratios were below 1.0 in all examined strata; however, most confidence intervals were wide and included 1.0. No statistically significant interaction was observed for labor analgesia, gestational diabetes mellitus and/or hypertensive disorders of pregnancy, or educational level. Given the small number of probable childbirth-related PTSD events within individual strata, these analyses had limited statistical power. Accordingly, the absence of statistically significant interactions should not be interpreted as evidence that the observed association was consistent across subgroups (**Supplementary Table 9**).

### 3.9 Exploratory comparison of elective and emergency cesarean deliveries

Among the 93 women who underwent cesarean delivery, 38 underwent an elective procedure and 55 underwent an emergency procedure. The mean City BiTS score was higher among women who underwent emergency cesarean delivery than among those who underwent elective cesarean delivery (20.2 ± 8.1 vs. 14.8 ± 6.9; P < 0.001). Mean PHQ-9 and GAD-7 scores were also higher in the emergency cesarean group. Probable childbirth-related PTSD was identified in 14 women (25.5%) in the emergency cesarean group and 6 women (15.8%) in the elective cesarean group; however, this unadjusted estimate was imprecise and did not reach statistical significance (P = 0.313). These descriptive findings should be interpreted cautiously because the two cesarean-delivery groups differed substantially in clinical indications, entry into labor, labor duration, fetal distress, and 1-min Apgar scores (**Supplementary Table 10**).

## 4 Discussion

This study provides real-world evidence that a structured, nurse-delivered psychological care pathway was associated with more favorable psychological and care-experience outcomes at 42 days postpartum among primiparous women. Compared with routine obstetric nursing alone, the structured program was associated with lower City BiTS scores, a lower prevalence of probable childbirth-related PTSD, fewer depressive and anxiety symptoms, and higher nursing satisfaction. A distinctive feature of this study is the evaluation of a standardized multicomponent pathway embedded within routine obstetric practice rather than an isolated psychotherapy session. The pathway combined universal core elements, including early psychological assessment, childbirth-related psychoeducation, emotional validation, guided relaxation, family-support guidance, intrapartum support, and early postpartum review, with need-based cognitive, mindfulness, and referral components. Its clinical relevance is supported by the documented intervention intensity, high completion of applicable core components, independent fidelity auditing, and limited between-nurse variation. The study also assessed childbirth-related trauma using both symptom severity and a complete Diagnostic and Statistical Manual of Mental Disorders, Fifth Edition–based classification with temporal attribution, providing a more clinically interpretable assessment than reliance on a single questionnaire cutoff. Collectively, these findings suggest that structured psychological nursing may represent a pragmatic framework for integrating trauma-informed assessment, supportive communication, and early recognition of psychological needs into routine maternity care (19, 20).

The trauma-related findings warrant interpretation at both the symptom-severity and classification levels. The 4.50-point between-group difference in the City Birth Trauma Scale score corresponded to a moderate standardized difference, whereas the prevalence of probable childbirth-related post-traumatic stress disorder was approximately halved in the psychological nursing group. The absolute risk difference of −8.63 percentage points further indicates that the observed contrast was potentially meaningful at the service-delivery level rather than reflecting statistical significance alone. The binary outcome was derived from the complete symptom-cluster, duration, impairment, exclusion, and temporal-attribution criteria, thereby distinguishing women with probable childbirth-related post-traumatic stress disorder from those with elevated symptoms that did not fulfill the full algorithm. The association between psychological nursing and lower odds of probable post-traumatic stress disorder remained after adjustment for delivery mode and maternal age and was similar in the Firth penalized analysis, suggesting that the estimate was not materially altered by small-sample bias within the parsimonious model. These findings are consistent with conceptual models in which childbirth-related post-traumatic stress arises through interactions among psychological vulnerability, perceived threat, reduced control, obstetric stress, and insufficient interpersonal support (21, 22). The components of the nursing pathway may be relevant to several of these processes: psychoeducation may reduce uncertainty, emotional validation may strengthen perceived support, and guided relaxation or mindfulness may assist with regulation of arousal. However, the program should be interpreted as an integrated care pathway rather than as evidence for the effect of any individual component. Delivery context also emerged as clinically important. Emergency cesarean delivery was associated with higher adjusted odds of probable post-traumatic stress disorder than vaginal delivery, while women undergoing emergency cesarean delivery had higher trauma, depressive, and anxiety symptom scores than those undergoing elective procedures. The unexpected nature of emergency intervention, prolonged labor, fetal distress, and reduced perceived control may contribute to this pattern (23, 24).

The findings for depressive and anxiety symptoms further support a multidimensional interpretation of postpartum psychological adaptation. The between-group differences in Patient Health Questionnaire-9 and Generalized Anxiety Disorder-7 scores were of small-to-moderate standardized magnitude, and the proportions of women screening positive for clinically relevant depressive or anxiety symptoms were lower by approximately nine percentage points in the psychological nursing group. These parallel associations are clinically plausible because childbirth-related post-traumatic stress, depression, and anxiety share several psychological features, including negative cognitions, heightened arousal, sleep disturbance, impaired emotional regulation, and reduced perceived coping capacity (25, 26). Nevertheless, the observed correlations were moderate to strong rather than complete: the correlation coefficients ranged from 0.58 to 0.72. This pattern suggests substantial shared variance while also supporting the interpretation of these measures as related but nonidentical domains (27, 28). However, these correlations do not indicate diagnostic equivalence, because each instrument assesses a distinct symptom domain. Moreover, as all three measures were administered at the same 42-day postpartum assessment, their temporal sequence and causal relationships could not be determined. From a clinical perspective, trauma-specific assessment may therefore identify difficulties that would not be fully captured by depression or anxiety screening alone, while combined assessment may provide a more comprehensive representation of postpartum psychological needs (29). The higher nursing satisfaction score in the psychological nursing group adds a complementary care-experience dimension. The moderate-to-large standardized difference may reflect greater attention to communication, respect, emotional responsiveness, continuity, and individualized support. Because these attributes are embedded within the nurse–patient relationship, satisfaction may represent not only approval of care delivery but also the extent to which women felt heard, informed, and supported during childbirth and early recovery. The coexistence of more favorable symptom profiles and higher satisfaction suggests that psychological care and perceived care quality may be closely interconnected within maternity services (30, 31). Exploratory subgroup analyses did not demonstrate statistically significant interaction by labor analgesia, pregnancy-related complications, or educational level. Accordingly, these analyses should be regarded as hypothesis-generating and do not establish that the observed associations were uniform across patient subgroups (32, 33).

The City BiTS was specifically developed to assess childbirth-related post-traumatic stress symptoms within the Diagnostic and Statistical Manual of Mental Disorders, Fifth Edition framework. Psychometric evaluation of the Italian version demonstrated satisfactory factorial validity, internal consistency, test–retest reliability, and convergent and predictive validity in a large postpartum sample (19, 28). Nevertheless, because the present classification was based on self-reported symptoms rather than a clinician-administered diagnostic interview, it should be interpreted as probable childbirth-related PTSD, not a confirmed diagnosis. Contemporary evidence indicates that childbirth-related PTSD reflects interactions among maternal vulnerability, obstetric stressors, perceived threat, loss of control, negative birth appraisal, and insufficient support (19, 29). The present nursing pathway addressed several potentially modifiable aspects of this framework through early assessment, psychoeducation, emotional support, relaxation training, family involvement, and referral when indicated. Meta-analytic evidence has similarly reported lower post-traumatic stress symptoms following structured early psychological interventions, although intervention content and study designs varied substantially (29). Thus, the lower City BiTS scores and prevalence of probable childbirth-related PTSD observed in our cohort are directionally consistent with previous evidence but do not establish intervention effectiveness. The higher odds of probable PTSD following emergency cesarean delivery further support closer psychological assessment after emergency obstetric intervention. Moreover, the reported co-occurrence of birth-related post-traumatic stress, depressive, and anxiety symptoms (30) accords with the moderate-to-strong correlations observed in our cohort and supports their interpretation as related but distinct dimensions of postpartum psychological health.

Several limitations should be acknowledged. First, this single-center retrospective temporal-cohort study used calendar-based rather than randomized allocation. Selection bias, residual confounding, and unmeasured temporal changes in obstetric practice, staffing, or nursing quality therefore cannot be excluded, and the findings should not be interpreted causally. Second, intervention fidelity was assessed retrospectively from routine documentation, and the program was an institutional clinical pathway rather than a fully manualized intervention, which may limit reproducibility and prevents attribution of the observed associations to individual components. Third, although outcome assessors were independent of intervention delivery, complete masking to group allocation could not be guaranteed or verified. Fourth, probable childbirth-related post-traumatic stress disorder was determined using a self-report DSM-5-based algorithm rather than a clinician-administered diagnostic interview. The institutional nursing satisfaction questionnaire also lacked external validation and an established minimal clinically important difference and was therefore treated as an exploratory care-experience measure. Fifth, assessment at a single 42-day postpartum visit precluded evaluation of symptom trajectories, delayed onset, and longer-term maternal or child outcomes. Finally, the limited number of probable post-traumatic stress disorder events reduced the precision of subgroup and interaction analyses. These limitations are balanced by several strengths, including evaluation of a standardized nurse-delivered pathway in routine obstetric practice, detailed documentation of intervention intensity and fidelity, complete psychological outcome data in the analytic cohort, and assessment of trauma using both symptom severity and a complete DSM-5-based algorithm with temporal attribution. Similar estimates from the Firth penalized analysis further supported the robustness of the primary model. Future multicenter randomized studies should incorporate standardized intervention manuals, prospective fidelity monitoring, blinded assessment, clinician-administered diagnostic interviews, validated patient-experience measures, longer follow-up, and larger samples to clarify subgroup differences and determine the optimal timing, intensity, and components of structured psychological nursing.

## 5 Conclusion

In conclusion, the addition of structured psychological nursing to routine obstetric care was associated with lower childbirth-related post-traumatic stress symptom severity, a lower prevalence of probable childbirth-related post-traumatic stress disorder, reduced depressive and anxiety symptoms, and higher nursing satisfaction at 42 days postpartum. After adjustment for delivery mode and maternal age, psychological nursing remained independently associated with lower odds of probable childbirth-related post-traumatic stress disorder. These findings suggest that structured psychological nursing may be a potentially valuable component of postpartum care, although prospective randomized studies are needed to confirm its effectiveness and generalizability.

## Data Availability

The raw data supporting the conclusions of this article will be made available by the authors, without undue reservation.
