# Supplementary material for: Structured psychological nursing and postpartum psychological outcomes in primiparous women: a retrospective temporal cohort study
Source: Front Psychiatry. 2026 Sep 10;17:1857487. doi: 10.3389/fpsyt.2026.1857487 (PMC13601254; doi:10.3389/fpsyt.2026.1857487)
Supplement: Supplementary file 1 [file Table1.docx]

Supplement Table S1. Missing-data profile and follow-up completion

| Variable or outcome | Eligible N | Missing, routine nursing group | Missing, psychological nursing group | Analyzed/evaluable N, routine nursing group | Analyzed/evaluable N, psychological nursing group | Reason for missingness | Handling method |
| --- | --- | --- | --- | --- | --- | --- | --- |
| Core demographic variables among included participants | 323 | 0/158 | 0/165 | 158 | 165 | None | Complete dataset |
| Pregnancy and obstetric variables among included participants | 323 | 0/158 | 0/165 | 158 | 165 | None | Complete dataset |
| Elective/emergency cesarean classification | 93 cesarean deliveries | 0/47 | 0/46 | 47 | 46 | None | Included as a three-level delivery-mode variable |
| Entry into labor | 323 | 0/158 | 0/165 | 158 | 165 | None | Complete dataset |
| Duration of labor among women entering labor | 291 | 0/143 | 0/148 | 143 | 148 | None | Analysis restricted to women entering labor |
| City BiTS item-level data at 42 days | 325 eligible for follow-up | 1/159 | 1/166 | 158 | 165 | One participant was unreachable and one declined follow-up | Complete-case analysis |
| PHQ-9 at 42 days | 325 | 1/159 | 1/166 | 158 | 165 | The same two participants did not complete the 42-day assessment | Complete-case analysis |
| GAD-7 at 42 days | 325 | 1/159 | 1/166 | 158 | 165 | The same two participants did not complete the 42-day assessment | Complete-case analysis |
| Nursing satisfaction questionnaire | 325 | 1/159 | 1/166 | 158 | 165 | The same two participants did not complete the 42-day assessment | Complete-case analysis |
| Component-level intervention-fidelity documentation | 165 | Not applicable | 8/165 | Not applicable | 157 | Incomplete component-level nursing records | Available-record analysis; no imputation |
| Intervention contact logs | 165 | Not applicable | 7/165 | Not applicable | 158 | Missing or incomplete contact-duration records | Available-record analysis; no imputation |
| Guided relaxation documentation | 165 | Not applicable | 3/165 | Not applicable | 162 | Relaxation checklist unavailable | Available-record analysis; no imputation |
| Family-support documentation among eligible mothers | 160 | Not applicable | 5/160 | Not applicable | 155 | Incomplete caregiver-support records | Available-record analysis; no imputation |
| Intrapartum-support documentation among women entering labor | 148 | Not applicable | 2/148 | Not applicable | 146 | Incomplete labor nursing records | Available-record analysis; no imputation |
| Postpartum-review documentation | 165 | Not applicable | 2/165 | Not applicable | 163 | Incomplete postpartum review records | Available-record analysis; no imputation |
| Cognitive-intervention documentation among eligible mothers | 67 | Not applicable | 4/67 | Not applicable | 63 | Incomplete cognitive-intervention records | Available-record analysis; no imputation |
| Mindfulness-exercise documentation among eligible mothers | 104 | Not applicable | 6/104 | Not applicable | 98 | Incomplete mindfulness records | Available-record analysis; no imputation |
| Additional-support documentation among eligible mothers | 31 | Not applicable | 2/31 | Not applicable | 29 | Incomplete follow-up support records | Available-record analysis; no imputation |
| Final primary-outcome analysis | 323 | 0/158 included participants | 0/165 included participants | 158 | 165 | All included participants had complete primary-outcome data | Complete-case analysis |
| Progressive muscle-relaxation documentation among eligible mothers | 155 | Not applicable | 0/155 | Not applicable | 155 | None | Complete available documentation |

Abbreviations: City BiTS, City Birth Trauma Scale; GAD-7, 7-item Generalized Anxiety Disorder scale; PHQ-9, 9-item Patient Health Questionnaire.

Supplement Table S2. Comparison of included and excluded participants

| Variable | Included participants, value (available N) | Excluded participants, value (available N) | Absolute SMD | P value |
| --- | --- | --- | --- | --- |
| Age, years | 28.8 ± 4.2 (n = 323) | 29.1 ± 4.5 (n = 31) | 0.069 | 0.724 |
| Gestational age at delivery, weeks | 39.2 ± 1.2 (n = 323) | 38.9 ± 1.5 (n = 30) | 0.221 | 0.295 |
| January–June delivery period, n/N (%) | 158/323 (48.9) | 15/31 (48.4) | 0.011 | 1.000 |
| Pre-delivery BMI, kg/m² | 26.2 ± 3.4 (n = 323) | 26.5 ± 3.8 (n = 24) | 0.083 | 0.710 |
| Any pregnancy-related complication, n/N (%) | 64/323 (19.8) | 7/29 (24.1) | 0.105 | 0.629 |
| Cesarean delivery, n/N (%) | 93/323 (28.8) | 10/30 (33.3) | 0.098 | 0.675 |
| Neonatal birth weight, g | 3344 ± 411 (n = 323) | 3290 ± 480 (n = 25) | 0.121 | 0.589 |
| 1-min Apgar score | 9.25 ± 0.65 (n = 323) | 9.10 ± 0.90 (n = 25) | 0.191 | 0.421 |

Gestational diabetes mellitus, hypertensive disorders of pregnancy, and other pregnancy-related complications were not mutually exclusive. Three women in the routine nursing group and two in the psychological nursing group had both gestational diabetes mellitus and a hypertensive disorder. Of the 23 women with other pregnancy-related complications, 15 also had gestational diabetes mellitus and/or a hypertensive disorder. Consequently, 64 unique women had at least one pregnancy-related complication.

Abbreviations: BMI, body mass index; SMD, standardized mean difference.

Supplement Table S3. Detailed intervention protocol and actual delivery

| Intervention component | Core/tailored | Recipient criteria | Planned timing | Planned frequency | Planned duration | Actual recipients | Actual contacts | Actual duration | Documentation source |
| --- | --- | --- | --- | --- | --- | --- | --- | --- | --- |
| Initial psychological assessment | Core | All mothers | Within 2 h after admission | Once | 10–15 min | Completed by 165/165 mothers (100.0%) | 165 | Median, 13 min | Admission psychological assessment form |
| Childbirth-related psychoeducation | Core | All mothers | Before active labor or cesarean delivery | Once | 15–20 min | Completed by 165/165 mothers (100.0%) | 165 | Median, 18 min | Nursing intervention checklist |
| Emotional validation and supportive communication | Core | All mothers | Pre-labor, intrapartum, and early postpartum periods | At least two contacts | 5–10 min/contact | All 165 mothers received at least one contact; 163/165 (98.8%) completed at least two contacts, whereas 2 received one documented contact | 501 | Median cumulative duration, 28 min | Nursing progress notes |
| Cognitive intervention | Tailored | Maladaptive beliefs, catastrophic thinking, or marked fear of childbirth | Before or during labor and early postpartum | One or two contacts | 15–20 min/contact | Of 67 eligible mothers, 61 completed the component (91.0%), 2 had documented noncompletion, and 4 had incomplete records; 98 were not eligible | 76 | Median cumulative duration, 22 min | Risk assessment and cognitive-intervention form |
| Breathing regulation | Core | All mothers unless clinically inappropriate | Before or during labor or before cesarean delivery | One guided session plus self-practice | 5–10 min | Among 162 mothers with evaluable records, 159 completed the component (98.1%) and 3 had documented noncompletion; records were unavailable for 3 additional mothers | 169 | Median, 8 min | Relaxation-training checklist |
| Progressive muscle relaxation | Tailored | Mothers able and willing to participate | Antepartum or early postpartum | Once; repeated when required | 10–15 min | Of 155 eligible mothers, 152 completed the component (98.1%) and 3 had documented noncompletion; 10 were not eligible | 154 | Median, 12 min | Relaxation-training checklist |
| Brief mindfulness exercise | Tailored | Elevated anxiety or psychological distress or participant preference | Antepartum or early postpartum | One or two sessions | 5–10 min/session | Of 104 eligible mothers, 89 completed the component (85.6%), 9 had documented noncompletion, and 6 had incomplete records; 61 were not eligible | 97 | Median cumulative duration, 10 min | Mindfulness exercise record |
| Family-support intervention | Core when an appropriate caregiver was available | Spouse or primary caregiver available | Antepartum or early postpartum | Once | 10–15 min | Of 160 eligible mothers, 151 completed the component (94.4%), 4 had documented noncompletion, and 5 had incomplete records; 5 had no available caregiver and were not eligible | 151 | Median, 12 min | Family-support record |
| Intrapartum emotional support | Core for women entering labor | Mothers who entered labor | During labor | As clinically required | 5–10 min/contact | Of 148 eligible mothers, 143 completed the component (96.6%), 3 had documented noncompletion, and 2 had incomplete records; 17 did not enter labor and were not eligible | 317 | Median cumulative duration, 20 min | Labor nursing record |
| Postpartum psychological review | Core | All mothers | At 24–72 h postpartum | Once | 10–15 min | Completed by 160 mothers; 3 had documented noncompletion and 2 had incomplete records | 160 | Median, 12 min | Postpartum psychological review form |
| Additional psychological support | Tailored | Persistent or worsening distress, elevated symptom scores, or nurse concern | Before discharge or during follow-up | As required | 15–30 min/contact | Of 31 eligible mothers, 29 received additional support (93.5%) and 2 had incomplete records; 134 were not eligible | 48 | Median cumulative duration, 30 min | Postpartum follow-up record |
| Specialist psychological or psychiatric referral | Tailored | Severe symptoms, marked functional impairment, self-harm concern, or persistent symptoms | At identification | Once, with subsequent follow-up | Not fixed | All 6 mothers meeting the referral criteria were referred (100.0%) | 6 referrals | Not applicable | Specialist referral record |
| Completion of all applicable core components | Core fidelity indicator | Mothers with complete component-level documentation | Across admission, delivery, and early postpartum periods | All applicable components | Target cumulative duration ≥60 min | Among 157 mothers with complete component-level documentation, 148 completed all applicable core components (94.3%) and 9 did not; documentation was incomplete for 8 mothers | Not applicable | Median total intervention duration, 95 min (IQR, 80–120 min) among 158 mothers with complete contact logs | Composite intervention-fidelity checklist |

All 152 mothers who completed progressive muscle relaxation had also completed breathing-regulation training; therefore, 159 unique mothers completed at least one guided relaxation component.

Abbreviation: IQR, interquartile range.

Supplementary Table S4. City Birth Trauma Scale DSM-5 scoring and temporal-attribution algorithm

| DSM-5 domain or scoring component | City BiTS items | Operational criterion | Coding or calculation rule |
| --- | --- | --- | --- |
| Stressor criterion A | Q1–Q2 | Exposure to perceived actual or threatened serious injury or death involving the mother or infant during childbirth | Code as fulfilled if Q1 or Q2 is answered “yes”; otherwise, code as not fulfilled |
| Re-experiencing criterion B | Q3–Q7 | Presence of at least one intrusion or re-experiencing symptom | Code as fulfilled if at least one item is scored ≥1; otherwise, code as not fulfilled |
| Avoidance criterion C | Q8–Q9 | Presence of at least one avoidance symptom | Code as fulfilled if at least one item is scored ≥1; otherwise, code as not fulfilled |
| Negative cognitions and mood criterion D | Q10–Q16 | Presence of at least two symptoms involving negative cognitions or mood | Code as fulfilled if at least two items are scored ≥1; otherwise, code as not fulfilled |
| Hyperarousal criterion E | Q17–Q22 | Presence of at least two hyperarousal symptoms | Code as fulfilled if at least two items are scored ≥1; otherwise, code as not fulfilled |
| Total symptom score | Q3–Q22 | Overall severity of the 20 DSM-5 PTSD symptoms | Sum the scores for Q3–Q22; possible total score range, 0–60, with higher scores indicating greater symptom severity |
| Dissociative subtype | Q23–Q24 | Presence of depersonalization or derealization symptoms | Record the dissociative subtype if either item is scored ≥1; this subtype was not required for the core probable PTSD classification |
| Symptom onset and temporal attribution | Q25 | Determine whether the reported PTSD symptoms began before or after the index childbirth | Q25 was not treated as a core DSM-5 diagnostic criterion. It was used to confirm that the symptoms did not predate the index childbirth and could therefore reasonably be attributed to the childbirth experience |
| Duration criterion F | Q26 | Persistence of symptoms for longer than 1 month | Code as fulfilled if Q26 is scored ≥1; otherwise, code as not fulfilled |
| Distress or impairment criterion G | Q27–Q28 | Presence of clinically relevant distress or functional impairment | Code as fulfilled if Q27 or Q28 is scored ≥1; otherwise, code as not fulfilled |
| Exclusion criterion H | Q29 | Symptoms potentially attributable to medication, alcohol, drugs, or physical illness | Code criterion H as present if Q29 is scored ≥1. Participants with criterion H present were excluded from the probable PTSD classification |
| Core probable PTSD classification | Q1–Q29 | Fulfillment of the DSM-5 stressor, symptom-cluster, duration, impairment, and exclusion requirements | Code as core probable PTSD if criteria A, B, C, D, E, F, and G are fulfilled and criterion H is absent |
| Probable childbirth-related PTSD classification | Q1–Q29, including temporal attribution from Q25 | Fulfillment of the core probable PTSD algorithm, with confirmation that symptoms began after and did not predate the index childbirth | Code as probable childbirth-related PTSD only if A=1, B=1, C=1, D=1, E=1, F=1, G=1, H=0, and Q25 does not indicate symptom onset before the index childbirth |
| Symptoms predating childbirth | Q25 | Core probable PTSD criteria fulfilled, but symptoms were reported to have been present before the index childbirth | Do not classify the participant as having probable childbirth-related PTSD; report separately as probable PTSD present at 42 days postpartum, where applicable |
| Uncertain or missing onset information | Q25 | Symptom onset relative to the index childbirth could not be established | Do not assign childbirth-related attribution in the primary analysis |
| Observed core probable PTSD cases | Complete Q1–Q29 algorithm | Number of participants fulfilling criteria A–G with criterion H absent | Routine nursing group: 28/158 (17.7%); psychological nursing group: 15/165 (9.1%); overall: 43/323 (13.3%) |
| Q25 indicating symptom onset after the index childbirth | Q25 among participants fulfilling the core probable PTSD algorithm | Symptoms began after the index childbirth | Routine nursing group: 28/28 (100.0%); psychological nursing group: 15/15 (100.0%); overall: 43/43 (100.0%) |
| Q25 indicating symptoms predating the index childbirth | Q25 among participants fulfilling the core probable PTSD algorithm | Symptoms were already present before the index childbirth | Routine nursing group: 0/28 (0.0%); psychological nursing group: 0/15 (0.0%); overall: 0/43 (0.0%) |
| Missing or uncertain Q25 onset information | Q25 among participants fulfilling the core probable PTSD algorithm | Symptom onset relative to childbirth was missing or uncertain | Routine nursing group: 0/28 (0.0%); psychological nursing group: 0/15 (0.0%); overall: 0/43 (0.0%) |
| Final probable childbirth-related PTSD cases | Complete DSM-5 algorithm plus Q25 temporal attribution | Core probable PTSD criteria fulfilled and symptoms confirmed not to predate the index childbirth | Routine nursing group: 28/158 (17.7%); psychological nursing group: 15/165 (9.1%); overall: 43/323 (13.3%) |

Notes: Q25 was used exclusively for temporal attribution and was not treated as an additional DSM-5 symptom or diagnostic criterion. Among the 43 participants who fulfilled the core probable PTSD algorithm, all reported that their symptoms began after the index childbirth. None reported symptoms predating childbirth, and none had missing or uncertain onset information. Therefore, all 43 cases fulfilling the core algorithm were retained in the final classification of probable childbirth-related PTSD.

The City BiTS total symptom score and the probable childbirth-related PTSD classification were analyzed as separate outcomes. Probable childbirth-related PTSD was not defined using a single total-score cutoff. Complete responses to the items required for the DSM-5 and temporal-attribution algorithms were required for classification.

Abbreviations: City BiTS, City Birth Trauma Scale; DSM-5, Diagnostic and Statistical Manual of Mental Disorders, Fifth Edition; PTSD, post-traumatic stress disorder.

Supplement Table S5. Nursing satisfaction instrument and psychometric assessment

Panel A. Instrument information

| Characteristic | Description |
| --- | --- |
| Instrument name | Institutional Nursing Satisfaction Questionnaire |
| Source | Developed and routinely used by the hospital nursing quality-management department |
| Version | 2024 obstetric nursing version |
| Number of items | 20 |
| Domains | Communication and respect; technical nursing care; emotional support; health education; continuity and responsiveness |
| Response format | Five-point Likert response |
| Item scoring | 1 = very dissatisfied to 5 = very satisfied |
| Total score range | 20–100; higher scores indicate greater satisfaction |
| Administration time | At the 42-day postpartum follow-up, after completion of the City BiTS, PHQ-9, and GAD-7 |
| Previously published external validation | None identified |
| Minimal clinically important difference | Not established |
| Interpretation | Exploratory patient-reported care-experience outcome rather than a validated clinical endpoint |

Panel B. Psychometric results

| Psychometric indicator | Overall cohort (n = 323) | Routine nursing group (n = 158) | Psychological nursing group (n = 165) |
| --- | --- | --- | --- |
| Questionnaire completion, n/N (%) | 323/323 (100.0) | 158/158 (100.0) | 165/165 (100.0) |
| Cronbach’s α | 0.91 | 0.89 | 0.92 |
| Floor effect, n (%) | 0 (0.0) | 0 (0.0) | 0 (0.0) |
| Ceiling effect, n (%) | 22 (6.8) | 6 (3.8) | 16 (9.7) |
| Mean total score | 89.3 ± 7.5 | 86.9 ± 7.5 | 91.6 ± 6.8 |

Abbreviations: City BiTS, City Birth Trauma Scale; GAD-7, 7-item Generalized Anxiety Disorder scale; PHQ-9, 9-item Patient Health Questionnaire.

Supplement Table S6. Predictor correlations and multicollinearity diagnostics

Panel A. Predictor correlation matrix

| Predictor | Psychological nursing group | Elective cesarean delivery | Emergency cesarean delivery | Maternal age |
| --- | --- | --- | --- | --- |
| Psychological nursing group | 1.000 | 0.011 | −0.035 | 0.024 |
| Elective cesarean delivery | 0.011 | 1.000 | −0.165 | 0.032 |
| Emergency cesarean delivery | −0.035 | −0.165 | 1.000 | 0.041 |
| Maternal age | 0.024 | 0.032 | 0.041 | 1.000 |

Panel B. Multicollinearity diagnostics

| Predictor | Tolerance | VIF | Conclusion |
| --- | --- | --- | --- |
| Psychological nursing group | 0.998 | 1.002 | No evidence of multicollinearity |
| Elective cesarean delivery | 0.971 | 1.030 | No evidence of multicollinearity |
| Emergency cesarean delivery | 0.969 | 1.032 | No evidence of multicollinearity |
| Maternal age | 0.996 | 1.004 | No evidence of multicollinearity |

Abbreviation: VIF, variance inflation factor.

Supplement Table S7. Firth penalized logistic regression sensitivity analysis for probable childbirth-related PTSD

| Predictor | Comparison/reference | Penalized adjusted OR | 95% profile-likelihood CI | P value |
| --- | --- | --- | --- | --- |
| Nursing strategy | Psychological nursing vs. routine nursing | 0.47 | 0.24–0.91 | 0.025 |
| Mode of delivery | Elective cesarean delivery vs. vaginal delivery | 1.29 | 0.51–3.15 | 0.580 |
| Mode of delivery | Emergency cesarean delivery vs. vaginal delivery | 2.77 | 1.30–5.93 | 0.008 |
| Maternal age | Per 1-year increase | 1.03 | 0.96–1.10 | 0.400 |

Abbreviations: CI, confidence interval; OR, odds ratio; PTSD, post-traumatic stress disorder.

Supplement Table S8. Spearman correlations among City BiTS, PHQ-9, and GAD-7 scores

| Variable | City BiTS | PHQ-9 | GAD-7 |
| --- | --- | --- | --- |
| City BiTS | 1.000 | ρ = 0.58 ; 95% CI, 0.50–0.65; P < 0.001; N = 323 | ρ = 0.61 ; 95% CI, 0.53–0.68; P < 0.001; N = 323 |
| PHQ-9 | ρ = 0.58 ; 95% CI, 0.50–0.65; P < 0.001; N = 323 | 1.000 | ρ = 0.72 ; 95% CI, 0.66–0.77; P < 0.001; N = 323 |
| GAD-7 | ρ = 0.61 ; 95% CI, 0.53–0.68; P < 0.001; N = 323 | ρ = 0.72 ; 95% CI, 0.66–0.77; P < 0.001; N = 323 | 1.000 |

Abbreviations: CI, confidence interval; City BiTS, City Birth Trauma Scale; GAD-7, 7-item Generalized Anxiety Disorder scale; PHQ-9, 9-item Patient Health Questionnaire.

Supplement Table S9. Exploratory subgroup analyses of probable childbirth-related post-traumatic stress disorder

| Subgroup | Stratum | Routine nursing group, events/N (%) | Psychological nursing group, events/N (%) | Unadjusted OR | 95% CI | P value | P for interaction |
| --- | --- | --- | --- | --- | --- | --- | --- |
| Labor analgesia among women who entered labor | Yes | 10/72 (13.9) | 5/81 (6.2) | 0.41 | 0.13–1.26 | 0.118 | 0.685 |
|  | No | 14/71 (19.7) | 8/67 (11.9) | 0.55 | 0.22–1.42 | 0.216 |  |
| Gestational diabetes mellitus and/or hypertensive disorders of pregnancy | Yes | 9/29 (31.0) | 5/27 (18.5) | 0.51 | 0.14–1.76 | 0.284 | 0.885 |
|  | No | 19/129 (14.7) | 10/138 (7.2) | 0.45 | 0.20–1.01 | 0.054 |  |
| Educational level | Junior college or below | 25/134 (18.7) | 13/137 (9.5) | 0.46 | 0.22–0.94 | 0.033 | 0.874 |
|  | Bachelor’s degree or above | 3/24 (12.5) | 2/28 (7.1) | 0.54 | 0.08–3.53 | 0.519 |  |

The labor-analgesia subgroup analysis was restricted to women who entered labor. Fifteen women in the routine nursing group and 17 women in the psychological nursing group did not enter labor and were excluded. These excluded participants included 4 and 2 probable PTSD events, respectively. Therefore, the labor-analgesia strata contained 24 and 13 events, whereas the full groups contained 28 and 15 events.

Abbreviations: CI, confidence interval; OR, odds ratio; PTSD, post-traumatic stress disorder.

Supplement Table S10. Characteristics and outcomes of elective versus emergency cesarean delivery

| Variable | Elective cesarean delivery (n = 38) | Emergency cesarean delivery (n = 55) | Effect estimate/absolute SMD | P value |
| --- | --- | --- | --- | --- |
| Age, years | 29.0 ± 4.2 | 29.3 ± 4.4 | SMD = 0.069 | 0.741 |
| Gestational age at delivery, weeks | 38.9 ± 1.1 | 39.2 ± 1.2 | SMD = 0.259 | 0.216 |
| Primary indication for cesarean delivery, n (%) |  |  | Cramér’s V = 0.685 | <0.001 |
| Malpresentation | 18 (47.4) | 4 (7.3) | — |  |
| Placenta previa or other antepartum indication | 10 (26.3) | 2 (3.6) | — |  |
| Fetal distress | 3 (7.9) | 23 (41.8) | — |  |
| Labor arrest or failed induction | 2 (5.3) | 21 (38.2) | — |  |
| Maternal request or other indication | 5 (13.2) | 5 (9.1) | — |  |
| Entered labor before delivery, n (%) | 6 (15.8) | 55 (100.0) | SMD = 3.266 | <0.001 |
| Duration of labor among women entering labor, h | 3.1 ± 1.5 (n = 6) | 10.1 ± 3.2 (n = 55) | SMD = 2.264 | <0.001 |
| Fetal distress, n (%) | 3 (7.9) | 23 (41.8) | SMD = 0.853 | <0.001 |
| Gestational diabetes mellitus, n (%) | 4 (10.5) | 8 (14.5) | SMD = 0.122 | 0.755 |
| Hypertensive disorders of pregnancy, n (%) | 3 (7.9) | 6 (10.9) | SMD = 0.103 | 0.733 |
| Postpartum hemorrhage, n (%) | 3 (7.9) | 5 (9.1) | SMD = 0.043 | 1.000 |
| Neonatal birth weight, g | 3390 ± 410 | 3290 ± 425 | SMD = 0.239 | 0.258 |
| 1-min Apgar score | 9.4 ± 0.5 | 9.0 ± 0.8 | SMD = 0.576 | 0.004 |
| City BiTS total symptom score | 14.8 ± 6.9 | 20.2 ± 8.1 | SMD = 0.707 | <0.001 |
| Probable childbirth-related PTSD, n (%) | 6 (15.8) | 14 (25.5) | SMD = 0.241 | 0.313 |
| PHQ-9 total score | 4.9 ± 3.0 | 6.7 ± 3.8 | SMD = 0.515 | 0.013 |
| GAD-7 total score | 4.5 ± 2.8 | 6.3 ± 3.5 | SMD = 0.557 | 0.007 |

For binary variables, SMDs were calculated as the difference in proportions divided by the square root of the average of the two group-specific binomial variances. Elective and emergency cesarean deliveries were classified according to the operative urgency documented in the contemporaneous clinical record. Recorded indications and intrapartum conditions were extracted separately and did not independently determine the urgency classification.

Abbreviations: City BiTS, City Birth Trauma Scale; GAD-7, 7-item Generalized Anxiety Disorder scale; PHQ-9, 9-item Patient Health Questionnaire; PTSD, post-traumatic stress disorder; SMD, standardized mean difference.
